# Improving the relevance of randomised trials to primary care: a qualitative study investigating views towards pragmatic trials and the PRECIS-2 tool

**DOI:** 10.1186/s13063-019-3812-7

**Published:** 2019-12-11

**Authors:** Gordon Forbes, Kirsty Loudon, Megan Clinch, Stephanie J. C. Taylor, Shaun Treweek, Sandra Eldridge

**Affiliations:** 1 Kings College London, London, UK; 2 University of Stirling, Stirling, UK; 3 Queen Mary, University of London, London, UK; 4 University of Aberdeen, Aberdeen, UK

**Keywords:** Pragmatic clinical trial, Primary health care, Randomised controlled trial, PRECIS-2

## Abstract

**Background:**

Pragmatic trials have been suggested as a way to improve the relevance of clinical trial results to practice. PRECIS-2 (Pragmatic Explanatory Continuum Indicator Summary-2) is a trial design tool which considers how pragmatic a trial is across a number of domains. It is not known whether a pragmatic approach to all PRECIS-2 domains leads to results being more relevant to primary care. The aim of this study was to investigate the views of people with influence on primary care practice towards the design of randomised trials, pragmatic approaches to trial design, and the PRECIS-2 domains.

**Methods:**

We carried out semi-structured interviews with people who influence practice in primary care in the UK. A thematic analysis was undertaken using the framework approach.

**Results:**

We conducted individual or small group interviews involving an elite sample of 17 individuals. We found that an exclusively pragmatic approach to randomised trials may not always make the results of trials more applicable to primary care. For example, it may be better to have less flexibility in the way interventions are delivered in randomised trials than in practice. In addition, an appropriate balance needs to be struck when thinking about levels of resourcing and the intensity of steps needed to improve adherence in a trial. Across other aspects of a trial’s design, for example the population and trial setting, a pragmatic approach was viewed as more appropriate.

**Conclusions:**

To maximize the relevance of research directed at primary care, trials should be conducted with the same populations and settings that are found in primary care. Across other aspects of trials it is not always necessary to match the conditions found in practice.

## Background

Randomised trials are seen by many as being the best design for providing evidence about the effectiveness of different interventions. However, they do not always produce evidence that is relevant to primary care because they are frequently conducted under conditions that are different from those found in primary care [1, 2]. In addition, primary care faces restrictions on resourcing [3] and a need for complex interventions, involving multiple interacting elements [4]; two factors that can further complicate the adoption of new interventions.

Pragmatic trials have been suggested as a solution to the problem of evidence not being relevant to clinicians, policymakers and patients. Pragmatic trials are often thought of as randomised trials that test interventions under the conditions found in routine care: aside from aspects of routine care modified by the intervention itself, other aspects of care should be as they usually would be [5–10]. Research funders including the National Institute of Health Research (NIHR) in the UK [11], the National Institute of Health (NIH) [12] and Patient Centred Outcome Research Institute (PCORI) [13] in the USA aim to fund pragmatic trials. There is growing interest in pragmatic trials from the pharmaceutical industry with the GetReal collaboration aiming to show how real world evidence, including pragmatic trials, could be used in pharmaceutical research and development [14].

**Fig. 1:**
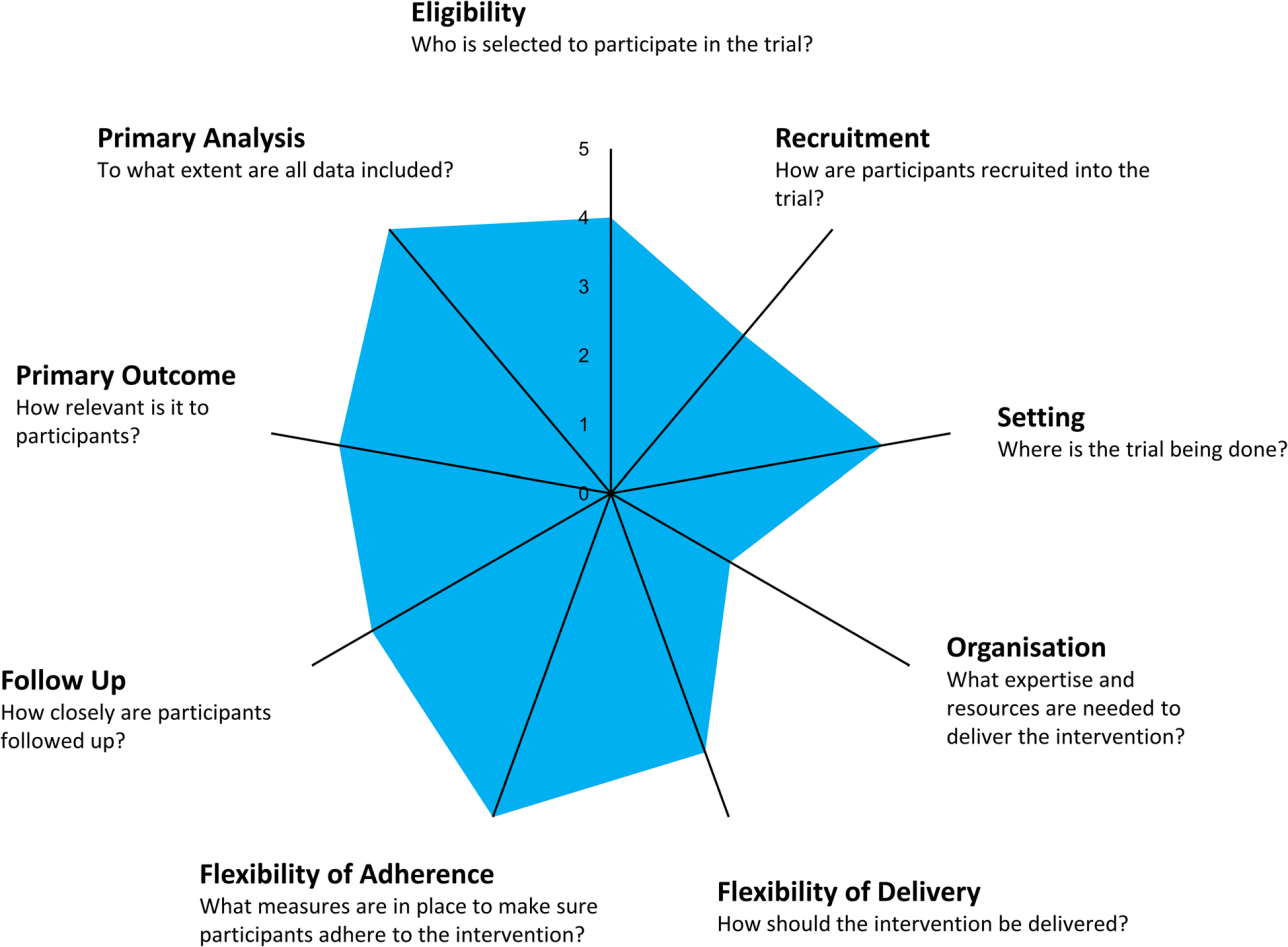
PRECIS-2 wheel for the COPERS [16], a largely pragmatic trial. For each domain higher scores indicate a more pragmatic approach and lower scores a more explanatory approach

When designing pragmatic trials, compromises often need to be made as routine care conditions cannot always be replicated within the trial. PRECIS-2 [15] (Fig. 1) is a trial design tool which has been developed to help make decisions about a trial’s design, by highlighting how pragmatic a trial is across nine different domains: eligibility, recruitment, setting, organisation, flexibility of delivery, flexibility of adherence, follow up, primary outcome, and primary analysis. The tool can be used to help investigators reflect on the design of their trial and ensure choices they have made allow the trial to achieve its goals. PRECIS-2 can be applied to individually randomised trials, cluster randomised trials [17] and also to systematic reviews [18].

To date, little research specific to primary care has been conducted to help those designing pragmatic trials make decisions to maximise the relevance of their results to practice. In particular we are aware of no research that considers the views of those implementing new research in this setting towards the compromises those designing trials may have to make. Previous research highlights challenges in defining complex interventions in pragmatic trials, deciding what steps should be taken to ensure compliance, and deciding the level of flexibility given to those delivering the intervention [1, 19, 20].

The aim of this study is to explore the views of stakeholders involved in influencing clinical practice in primary care towards the design of randomised trials. We investigate how evidence from trials is used and which aspects of a trial’s design influence how the results are interpreted. General views about pragmatic trials are explored and the PRECIS-2 tool is used to facilitate a discussion about specific decisions researchers can take to maximise the relevance of their trials to primary care.

## Methods

We conducted individual and small group interviews to investigate the views of people involved in influencing primary care practice on how best to design trials and on views towards areas of design covered by the PRECIS-2 domains. We sampled from groups who had a professional interest in the wider dissemination of research findings into primary care practice.

Through research team discussion and consultation with academics in primary care, we identified seven categories of people who used the results of primary care trials to influence practice, and aimed to include people from each category in our sample. The different categories were journal editors, primary care educators, guideline developers, research charities, research funders, clinical commissioning group leads and quality improvement organisations. Research funders were included as their role in deciding what research gets carried out has considerable implications for how clinical practice evolves. Research charities were included as a category due to their work in funding research and because of the work they do advocating for changes in health policy and clinical practice.

We identified individuals from each category either from appropriate websites or through personal networks of the research team and approached via email. The invitation email described the work as a “study investigating how best to design and conduct clinical trials in primary care so that they deliver results that are relevant to practice” and informed potential participants that we would be using PRECIS-2 and provided a brief description of the tool. We invited participants until we had at least one person from each category. We invited more than one person in each category at a time to maximise recruitment; if more than one person from a category responded to our invitation we interviewed all of them. The sample size was limited as the study targeted an elite group of stakeholders tasked with the job of synthesising and rolling out research evidence.

The interviews were carried out by GF (Gordon Forbes), either face to face or via video link (Skype). Face to face interviews were carried out at the participants place of work or in another location of their choosing. The interviews were scheduled to last 1 h and lasted between 45 min and 1 h 15 min. At the time of the interviews GF was a male, research methods fellow who had received training in qualitative research methods. For two interviews GF was joined by a male academic GP who co-led the interview. The academic GP had received training in qualitative research and had experience conducting qualitative research. All participants were unknown to the interviewers prior to the interviews taking place.

GF (Gordon Forbes) developed the topic guide for the interviews through discussions with the research team. This guide was refined throughout the research process, for example some vignettes of trials used in early interviews were dropped from later interviews because discussion of these vignettes left little time for discussion of other important topics. The final topic guide is reported in Table 1.

**Table 1:** Topic guide

• Interviewer introduction—GF introduced himself and the study. He introduced himself as a researcher interested in pragmatic trials, describing the research as a study into how best to design primary care trials so that they are relevant to practice. PRECIS-2 is mentioned in passing but not the details of how the tool works. Where the academic GP was present they were introduced as a GP with research interests similar to GF	
• Participant(s) introduction	
• How participants use evidence from randomised trials	
• What aspects of trials do participants think are important when deciding whether research is relevant to practice	
• PRECIS-2 is introduced by the interviewer with the participant shown an example PRECIS-2 wheel. The concepts of pragmatic and explanatory trials are also explained	
• How important is it that research is similar to routine practice for each of the PRECIS domains for the results of the trial to be relevant to practice?	
• Closing. Interviewee(s) is asked if there is anything else they would like to say or comment on and given the opportunity to ask any questions they have about the study	

Prior to the interviews participants provided consent to participate in the study. Interviews were recorded and transcribed by an independent transcription service. One interview was transcribed by GF. Transcripts and findings of the study were not returned to participants for comment.

A thematic analysis was carried out following the Framework method [21]. Familiarisation was carried out by four of the study authors (GF, SE (Sandra Eldridge), KL (Kirsty Loudon) and MC (Megan Clinch)): GF read all of the interview transcripts and notes. SE, KL and MC each read a different subset so that all the interview transcripts were reviewed by two different people. Analysis was conducted using NVivo version 10. A thematic framework was developed by GF and reviewed with SE, KL and MC. Coding was conducted by GF and codes were reviewed and discussed with SE, KL and MC. Indexing and Charting were carried out by GF and reviewed by SE, KL and MC. Interpretation of the results was carried out by all authors of the study.

## Results

We sent 24 invitations to groups or individuals to participate in the study; we conducted 12 interviews and 12 invitations were declined. Seventeen individuals took part in total, nine via individual interviews and eight via group interviews (Table 2). Interviews were conducted between October 2014 and February 2015. Three of the individual interviewees did not give permission for their interviews to be recorded. For two interviews the recording equipment failed so in total seven interviews (two group and five individual) were recorded and transcribed. We used contemporaneous field notes from the remaining five interviews.

**Table 2:** Summary of interviews

Interview	Number of Interviewee(s)	Role	Primary Care Clinician
Interview A*	3	Guideline developer	No
Interview B	1	Guideline developer	No
Interview C*	1	Guideline developer	No
Interview D	2	Primary care support and quality improvement	Yes
Interview E	1	Primary care educator	Yes
Interview F	1	Primary care commissioner	Yes
Interview G	3	Research charity (two members of research funding team, one member of research policy team)	No
Interview H^†^	1	Chair of public funding panel	Yes
Interview I^†^	1	Chair of public funding panel	No
Interview J^†^	1	Health technology assessment	No
Interview K^†^	1	Editor of journal publishing systematic reviews	No
Interview L^*†*^	1	*Clinical director of journal publishing systematic reviews*	No

*Academic GP co-lead interview

^†^Only field notes available for interview

Four main themes emerged from our interviews: how evidence is used; aspects of trials considered when assessing evidence; views on PRECIS-2 domains; and perceptions of pragmatic and explanatory trials.

### How evidence is used

Interviewees who applied evidence from randomised trials in a clinical setting reported using evidence differently from those who produced evidence synthesis or guidelines. The latter followed a formal process starting with a systematic way of identifying evidence, critical appraisal and then production of a summary of the evidence as a review or guideline. Challenges in applying evidence came from combining the results of heterogeneous evidence and deciding whether evidence is applicable to the question of interest. In overcoming these challenges judgement has to be incorporated into the formal processes. The greatest area of judgement was around whether evidence is applicable to the question of interest.

*“[the guideline development group members] make, what we call, a considered judgement on evidence from the trials, taking into account other aspects, whether it’s generalizable, whether it’s applicable.”*

(Interview C, *Guideline developer*)

For those applying evidence in a clinical setting, sources of evidence included guidelines or evidence summaries as well as less formal sources such colleagues or experts in a particular field. Due to time pressure and a need for accountability regarding decisions about patient care, the results from single studies were not commonly used to influence practice. We found a mistrust of early evidence due to the potential of trials to show greater benefit of new interventions than would be found in practice. To overcome this challenge some individuals employed a deliberate strategy of waiting before implementing new evidence. A further challenge in applying evidence was the limited resources to implement novel interventions.

*“So most of the time, what you’ll find is on my computer screen [is], NICE [The National Institute for Health and Care Excellence] or CKS [Clinical knowledge summaries] open up in a separate window, that I’ll just refer to if I need to with every patient.”*

(Interview E, Primary care educator)

“*…let’s just take the new oral anticoagulant agents … There’s been some early meta-analyses, but everybody feels the early trials were always more optimistic…*

(Interview D, *Primary care support and quality improvement*)

“*…the question is, do you wish to be an early adopter, are the advantages so great that you want to take a risk or would you like to do it later.*”

(Interview D, *Primary care support and quality improvement*)

Interviewees from the research charity were involved in advocating for change based on the results of new research and facilitating discussions amongst clinicians of issues arising in new evidence. They focused on trials funded by their charity. Rather than applying a formal process of critical appraisal they appeared to try to ensure quality by funding research meeting certain standards.

“*And we also use [examples of clinical trials funded by the charity] quite heavily in case studies when we’re talking to government, just as examples of how charities operate in the UK and demonstrations of exciting new things that are happening.*”

(Interview D, *Primary care support and quality improvement*)

The funders interviewed provided perspectives on the use of evidence but from their roles as clinicians and as research funders. From the perspective of funders they advocated that research be disseminated via systematic reviews and guidelines. As clinicians their views towards evidence use were consistent with those already presented.

“*Systematic reviews of all relevant research should be carried out and these should be disseminated and used to change practice. This is where organisations such as NICE can play an important role in producing guidelines based on the best evidence.*”

(Interview I, Research funder)

### Perceptions of pragmatic trials

Interviewees perceptions of pragmatic trials included enthusiasm, mistrust alongside limited knowledge of the term and misconceptions about its meaning. Interviewees from public research funders and journals were most enthusiastic about pragmatic trials, showing existing familiarity with the concept and positively expressing that those are the sort of trials they would be most interested in.

“*We are more interested in funding pragmatic trials than explanatory and would like to see trials which were as pragmatic as possible...*”

(Interview H, Research funder)

Interviewees from the research charity, involved in primary care education or from CCGs, and some interviewees involved in guideline development had little pre-existing knowledge of the term “pragmatic trials”, expressing misconceptions as to what the concept meant or it being a term they were not familiar with prior to the interview. Misconceptions about pragmatic trials included conflating the design with trials of complex interventions and with particular design features such as cluster randomisation or open label studies. It was also suggested that pragmatic trials would only be relevant to in particular clinical areas. Some interviewees also drew a distinction between pragmatic trials and randomised controlled trials.

*“...to include [pragmatic trials] the guideline developers would have to be really careful to explain the difference between this and RCT [randomised controlled trial].”*

(Interview C, *Guideline developer*)

Amongst those who used evidence from guidelines or evidence synthesis, pragmatic trials were welcomed as they simplified the judgement around whether evidence is applicable to clinical settings. There was concern, however, that they could be significantly different from trials in the existing evidence base leading to heterogeneity which could make meta-analysis more difficult.

“*Pragmatic trials may have to be considered separately in meta-analysis due to heterogeneity with other trials*”

(Interview J, Health technology assessor)

“*I can just imagine that people would be really thrilled to see a pragmatic trial, of diabetes or something, [set here] and that people could then use it... It would be so much better for recommendations.”*

(Interview C, *Guideline developer*)

Pragmatic trials also carried negative connotations, sometimes being viewed as inferior to more traditional approaches to randomised trials due to perceived weaknesses.

“*Pragmatic can be a dirty word when describing trials, people like to shoot at them…*”

(Interview K, Editor of Journal publishing systematic reviews).

There was also concern that the degree of pragmatism of trials could be used to manipulate the systematic review process.

“*During discussions of evidence it can be difficult to consider the relative pragmatism of evidence—sometimes it is used by people to try and exclude evidence that doesn’t agree with their point.*”

(Interview I, Research funder)

### Views on the PRECIS-2 domains

The population in the trials was by far the most discussed aspect of a trial’s design, with interviewees preferring trial populations to have few exclusions, including patients with comorbidities and older patients. Population is handled by PRECIS-2 over three domains: eligibility, recruitment and setting (Fig. 1).

*“So again, if you do it in your tertiary centres, then it becomes almost inapplicable in primary care, because I don’t have those resources. I don’t see all of those patients at that stage in that illness. I see them either way before or way after they’ve seen the tertiary care people. So yes I think setting is very important.”*

(Interview E, *Primary care educator*)

Aside from its part in determining the population in trials, recruitment was an aspect of trial design not explicitly considered by most interviewees when assessing evidence. Amongst those who did consider the impact of recruitment on the relevance of trial results, there was concern that very intensive recruitment could lead to people being included in trials who would not usually present for treatment in routine practice.

“*…we wouldn’t assess recruitment routinely, but it’s about whether recruitment is applicable to the question that we‘re trying to address.*”

(Interview D, *Guideline developer*)

*“Sometimes recruitment can be too intensive and bring people into the trial who would not usually present for a condition”*

(Interview I, *Chair of funding panel*)

The organisation domain focuses on the level of expertise and resources made available to deliver an intervention compared with what would be available in practice. Here, we identified a tension between a pragmatic and an explanatory approach. Those applying research in practice were more concerned about resourcing issues and preferred trials to test interventions that could be implemented with the limited resources available in primary care. Those from the research charity or guideline developers believed there was also a place for evidence from trials of interventions requiring resources over and above those currently available. They felt that sometimes research showing that a resource-intensive intervention was effective could lead to those resources becoming available in routine care.

*“The only thing in primary care that would be a limiting factor is the resources aren’t this sort of overflowing bucket”*

(Interview E, *Primary care educator*)

*“And sometimes groups will make the gold standard recommendation and that will push forward what resources are brought in”*

(Interview C, *Guideline developer*)

For flexibility of delivery interviewees producing evidence synthesis or guidelines favoured reduced flexibility as this allows more understanding to be gained as to what is causing any effect, makes it easier to include a trial in a meta analysis and also reduces bias from other treatments being initiated. Reduced flexibility was also preferred as it allowed greater understanding of what the intervention being delivered in the trial actually is. For the clinicians interviewed there was an appreciation and expectation that trials would have less flexibility in the way interventions were delivered.

“ *After ten years of doing this I would prefer to see strict control [in how interventions are delivered] but if there are variation they need to be described properly so in attempting to make sense of this you can see what has happened.*”

(Interview B, *Guideline developer*)

“*We appreciate that you’ve got to stick to strict guidelines when you’re doing the research, otherwise it doesn’t become very accurate at the end of it. So as long as it’s not hugely different, we appreciate there is a little bit of leeway in real life, but we wouldn’t expect that in a clinical trial.*”

(Interview E, *Primary care educator*)

Poor adherence to interventions in trials was a concern as this can reduce the potential effect of a successful intervention. For some, best practice with regard to flexibility of adherence was for adherence issues to be identified prior to the trial and in the trial itself no extra steps to be taken to improve adherence. Others, notably the interviewees from the research charity, suggested knowing an intervention can be effective can lead to measures to help adherence being developed.

“*Researchers shouldn’t include intense follow-up to ensure adherence. Steps should be taken to collect as much primary end point data as possible but this is separate to ensuring that people adhere to the intervention.*”

(Interview I, *Chair of funding panel*)

“*The more flexibility there, the more you’re going to actually mask any real effect because of the amount of variation... ...find out why people aren’t adhering and what can we do to try and help people adhere to that particular exercise programme, for example. So, you know, I feel that the flexibility [of adherence] should be pushed tighter.*”

(Interview G, Research funding and policy, Research charity)

For follow-up we identified a balance to be struck between collecting data that are useful for research and follow-up influencing participant behaviour or increasing the burden on those taking part. There was also concern raised by some interviewees that in some settings intensive follow-up could act as an additional intervention.

“ *...in terms of having quite a lot follow up, you can get some really useful answers and actually you probably want to do that. But it’s thinking about the way in which you follow-up and so that you’re not actually influencing their behaviour and their clinical outcome through taking those measurements.*”

(Interview G, Research funding and policy, Research charity )

Where primary outcome and primary analysis were discussed all interviewees favoured a more pragmatic approach with patient-centred primary outcomes and intention to treat analysis. There was concern expressed about primary outcomes being measured at too early a time point.

#### Comments on applying PRECIS-2

The guideline developers, research charity and research funders considered PRECIS-2 a useful tool, with the guideline developers saying that it covered many of the areas of judgement they were required to make and those involved in research funding commenting that being able to justify design decisions across the PRECIS-2 domains would strengthen funding applications.

“*we never used this instrument, it looks very helpful, we were often left to make the judgement... to what degree was it moving towards the pragmatic, and to what degree was it explanatory.*

(Interview B, *Guideline Developer*)

“*if they thought about all of them in advance and they’ve got a good reason why it’s explanatory on this and it’s pragmatic on this one, then I think that’s, that would make a strong application coming through.*”

(Interview G, Research funding and policy, Research charity)

Alternative uses for PRECIS-2 were also suggested, including use as a teaching aid, trial reporting, and aiding judgements around applicability. It was noted that PRECIS-2 is subjective so care would have to be taken for reported PRECIS-2 scores to be justified.

“*…actually in the paper they should put [PRECIS-2 wheels] in… you just want a quick summary. That might be helpful*”

(Interview F, *Primary care commissioner*)

#### Issues raised by participants but not covered by PRECIS-2 domains

Outside of PRECIS-2 domains, interviewees raised issues around internal validity, in particular blinding where possible, and size of the trial, with larger trials preferred. Internal validity, sometimes referred to by interviewees as “quality”, was typically assessed before generalisability when developing guidelines or assessing research for funding, typically using risk of bias tools. Issues around internal validity were raised both by those involved in evidence synthesis and by those applying evidence to practice.

“*So if the quality is poor, that will get marked before people even think whether it’s generalisable or not.*”

(Interview C, *Guideline developer*)

Of the factors unrelated to the design of trials, reporting of the trial was the most important issue outside PRECIS-2 domains to be raised. Poor reporting was seen as an obstacle to using evidence in practice whereas good reporting was seen as something which could enhance the generalisability of evidence. Areas of reporting that were most important included details of what the intervention was, how the intervention was implemented in the trial and discussion of the generalisability of results. If usual care is used as a comparator, it was seen as important to report in detail what usual care consisted of.

“*I think we’ve found that people have not been able to use evidence before because they haven’t been clear on what the usual care has been.*”

(Interview C, *Guideline developer*)

Other issues that were raised included patient acceptability of the intervention, whether research was carried out in collaboration with practice and whether the intervention addressed an important clinical problem either effecting a large number of people or a particular problem for a minority that has been hard to reach.

## Discussion

### Summary

Whilst broadly supporting the principle of pragmatic trials, this study identifies a number of issues that those conducting and funding trials of interventions to be delivered in primary care should take into account to improve the relevance of the research to primary care. The term “pragmatic trial” is not universally recognised and sometimes misunderstood. Whilst pragmatic trials were welcomed by some of our interviewees, others showed less familiarity with the concept or expressed suspicion towards trials labelled as pragmatic due to a perceived lack of rigour.

Across the PRECIS-2 domains eligibility, setting, primary outcome and primary analysis the universal response from our interviewees was that more pragmatic trial designs would make results most useful. In particular including the same population as would present in practice, having patient centred outcomes and conducting intention to treat analysis.

For the domains recruitment, flexibility of adherence, organisation, follow-up, a balance needs to be struck between testing the intervention under more restricted conditions and a more pragmatic approach. For recruitment and follow up, it was acknowledged by some interviewees that for trials to be successful they cannot mirror routine care, and a slightly less pragmatic approach could be necessary. However, extreme departures from routine care for follow-up or recruitment were discouraged, in particular for follow-up where interviewees were concerned about intensive follow-up influencing behaviour.

For organisation and flexibility of adherence there was a tension between recognising the limitations that an intervention will encounter in everyday practice and providing results that can lead to change. Showing an intervention is effective when delivered with greater resources or expertise than can be found in practice could lead to better resourcing being made available. Similarly, showing that high adherence to an intervention leads to better outcomes could motivate efforts to improve adherence to treatments. There was a contrast in responses between the clinicians interviewed and some of our other interviewees, most notably those from the research charity. The clinicians generally favoured a more pragmatic approach, taking into account the constraints of the system in which they worked. Interviewees from the research charity and involved in guideline development saw a place for designs that are less pragmatic in terms of organisation or flexibility of adherence as these trials may provide evidence that leads to system level. It is worth noting that delivering interventions with greater levels of resources than can be found in practice may be challenging with some public funders as research funding does not necessarily cover excess treatment costs [22].

For flexibility of delivery a less pragmatic approach was favoured. Being able to clearly identify the intervention delivered in a trial was more important to our participants than trying to reproduce in the trial the amount of flexibility that would exist in practice when delivering interventions.

Our findings relating to how evidence is used by clinicians are not new and have been explored in more detail by others [23]. It is worth noting, however, that clinicians routinely accessed evidence from clinical trials via guidelines, highlighting the importance of trials being conducted in a way that is amenable to the guideline development process. One of the key challenges identified for those developing guidelines is assessing whether evidence is applicable. In addition, good reporting, particularly details of the intervention, can enhance the applicability of a trials results. Steps taken to maximise internal validity are also important, for example blinding, as this is often assessed before the applicability of trials is considered.

### Strengths and limitations

This study sought the views of people from a variety of backgrounds involved in applying evidence from randomised trials towards the design of pragmatic trials, obtaining a wide range of views highlighting aspects of design where there is consensus and areas where decisions are more contentious. The study included a limited elite sample and was not designed to achieve saturation. There was evidence of saturation across the themes of “How evidence is used”, “aspects of trials considered when assessing evidence” and “views on the PRECIS-2 domains”, with our later interviews bringing limited new views. For our fourth theme, “perceptions of pragmatic and explanatory trials”, we found a wide range of views and whilst there was repetition of some of the key ideas we cannot be certain saturation was reached.

The researchers conducting the study presented themselves as researchers involved in carrying out pragmatic trials, so more critical views of pragmatic trials may not have been encountered. Whilst the sample did not include primary care clinicians as a separate category a number of clinicians were included, ensuring that their contributions are well represented. All stages of data analysis involved at least four different researchers, helping to avoid the interpretation of the results being dependent on the interpretation of a single person.

Discussing PRECIS-2 in interviews presented challenges as it was only possible to convey a relatively superficial level of understanding of the tool. On the other hand the use of PRECIS-2 enabled a detailed discussion of the specifics of pragmatic trial design, without relying on the interviewees’ understanding of what it means for a trial to be pragmatic.

### Comparison with existing literature

This study is the first to examine pragmatic trials from the point of view of those funding and disseminating evidence for primary health care. Too much flexibility in the way interventions are delivered has been identified as posing problems in pragmatic trials in three previous studies [24–26]. These studies raise concerns about the challenges too much flexibility may present to those delivering the intervention [25] and identify safety concerns when implementing a new intervention without strict guidelines [26]. The tension between fidelity to the intervention and its delivery and flexibility so that the intervention can be implemented widely is also described in implementation research [27]. The trade-off between testing interventions within the resource constraints found in practice, and conducting trials that can lead to better resources in practice, has not received much attention previously in the pragmatic trial literature. The need for enhanced descriptions of interventions has been identified for complex interventions [28] and is highlighted in the consort extension for pragmatic trials [8], and the TIDiER checklist for reporting interventions [29].

### Future research

Further work in this area could explore whether the findings of this study are generalisable to other clinical settings. Work to improve adherence to existing reporting guidelines [8, 29] could also improve the generalisability of trial results in primary care. Closer collaboration between trialists conducting pragmatic trials and people involved in evidence synthesis and guideline development could help ensure new trials fit smoothly into the guideline development process.

## Conclusions

Funders and trialists investigating interventions that will be applied in primary care should fund and conduct randomised trials that are pragmatic in terms of the population included in the trial, the setting and trial outcomes. Particular care should be taken in the areas of trial design highlighted here by our interviewees (recruitment, organisation, flexibility of adherence, flexibility of delivery, and follow-up) where a completely pragmatic approach may not be best. The lack of universal understanding of the term “pragmatic trial” shows a need for promoting better understanding of pragmatic trials and a need for those conducting pragmatic trials to be explicit about how their trial is, and is not, pragmatic. The PRECIS-2 tool can assist with defining how pragmatic a trial is and could be used to help people understand what it means for a trial to be pragmatic. Good reporting of trials is important for ensuring their applicability, particularly details of the intervention delivered and what constitutes usual care in the clinical setting in which the intervention is being evaluated.

## Data Availability

Anonymised study data are available upon reasonable request. Please contact pctu-data-sharing@qmul.ac.uk with any data sharing requests.

## Abbreviations

CKS: Clinical knowledge summaries
NICE: The National Institute for Health and Care Excellence
NIH: National Institute of Health
NIHR: National Institute of Health Research
PCORI: Patient Centred Outcome Research Institute
PRECIS-2: Pragmatic Explanatory Continuum Indicator Summary-2
RCT: Randomised controlled trial
